# A strategy to reduce the false‐positive rate after low‐dose computed tomography in lung cancer screening: A multicenter prospective cohort study

**DOI:** 10.1002/cam4.6106

**Published:** 2023-05-18

**Authors:** Zheng Wu, Fengwei Tan, Yaozeng Xie, Wei Tang, Fei Wang, Yongjie Xu, Wei Cao, Chao Qin, Xuesi Dong, Yadi Zheng, Zilin Luo, Chenran Wang, Liang Zhao, Changfa Xia, Jiang Li, Renda Li, Feiyue Feng, Jibin Li, Jiansong Ren, Jufang Shi, Hong Cui, Sipeng Shen, Ning Wu, Wanqing Chen, Ni Li, Jie He

**Affiliations:** ^1^ Office of Cancer Screening, National Cancer Center/National Clinical Research Center for Cancer/Cancer Hospital, Chinese Academy of Medical Sciences and Peking Union Medical College Beijing China; ^2^ Department of Thoracic surgery, National Cancer Center/National Clinical Research Center for Cancer/Cancer Hospital Chinese Academy of Medical Sciences and Peking Union Medical College Beijing China; ^3^ The Second People's Hospital of Liaocheng Shandong China; ^4^ Chinese Academy of Medical Sciences Key Laboratory for National Cancer Big Data Analysis and Implement Chinese Academy of Medical Sciences and Peking Union Medical College Beijing China; ^5^ Department of Epidemiology, Center for Global Health School of Public Health, Nanjing Medical University Nanjing China; ^6^ Jiangsu Key Lab of Cancer Biomarkers, Prevention and Treatment, Collaborative Innovation Center for Cancer Personalized Medicine Nanjing Medical University Nanjing China; ^7^ PET‐CT Center, National Cancer Center/National Clinical Research Center for Cancer/Cancer Hospital Chinese Academy of Medical Sciences and Peking Union Medical College Beijing China; ^8^ Department of Diagnostic Radiology, National Cancer Center/National Clinical Research Center for Cancer/Cancer Hospital Chinese Academy of Medical Sciences and Peking Union Medical College Beijing China

**Keywords:** early diagnosis and early treatment, lung cancer, prediction model, pulmonary nodules, screening

## Abstract

**Background:**

The ability of lung cancer screening to manage pulmonary nodules was limited because of the high false‐positive rate in the current mainstream screening method, low‐dose computed tomography (LDCT). We aimed to reduce overdiagnosis in Chinese population.

**Methods:**

Lung cancer risk prediction models were constructed using data from a population‐based cohort in China. Independent clinical data from two programs performed in Beijing and Shandong, respectively, were used as the external validation set. Multivariable logistic regression models were used to estimate the probability of lung cancer incidence in the whole population and in smokers and nonsmokers.

**Results:**

In our cohort, 1,016,740 participants were enrolled between 2013 and 2018. Of 79,581 who received LDCT screening, 5165 participants with suspected pulmonary nodules were allocated into the training set, of which, 149 lung cancer cases were diagnosed. In the validation set, 1815 patients were included, and 800 developed lung cancer. The ages of patients and radiologic factors of nodules (calcification, density, mean diameter, edge, and pleural involvement) were included in our model. The area under the curve (AUC) values of the model were 0.868 (95% CI: 0.839–0.894) in the training set and 0.751 (95% CI: 0.727–0.774) in the validation set. The sensitivity and specificity were 70.5% and 70.9%, respectively, which could reduce the 68.8% false‐positive rate in simulated LDCT screening. There was no substantial difference between smokers' and nonsmokers' prediction models.

**Conclusion:**

Our models could facilitate the diagnosis of suspected pulmonary nodules, effectively reducing the false‐positive rate of LDCT for lung cancer screening.

## 1 BACKGROUND

Lung cancer is one of the most common and deadliest malignant tumors worldwide and in China.^1^, ^2^ In 2020, the number of new cases of lung cancer in China reached 815,563, and 714,699 patients died of lung cancer.^2^ The survival rate of stage IV lung cancer is close to 0, whereas that of stage I lung cancer is up to 80%.^3^ Therefore, it is essential to identify patients at an early stage of lung cancer to improve their prognosis.

Screening with low‐dose computed tomography (LDCT) is recognized as an effective way to reduce the lung cancer disease burden and has been demonstrated to decrease lung cancer mortality rates by 20%–24%^4^, ^5^; our team also assessed the effect of lung cancer screening in Chinese population, which demonstrated that one‐off LDCT scan access would reduce 31% of the mortality of lung cancer in the target population.^6^ However, the false‐positive rate (FPR) of LDCT in lung cancer screening could be as high as 96.4%^4^; patients with benign nodules who undergo unnecessary diagnostic procedures face radiation exposure, medical expenses, and physical or mental burdens.^7^, ^8^, ^9^ Therefore, decreasing the FPR of LDCT scans is crucial.

The early manifestations of lung cancer are nodules, and the imaging characteristics of pulmonary nodules can often directly reflect the lung lesions. Using patient information, such as epidemiological factors, and imaging characteristics of pulmonary nodules, prediction models can predict and evaluate the risk of lung cancer in patients with positive LDCT screening. International guidelines recommend the use of prediction models to reduce the FPR of screening.^10^, ^11^ However, our previous systematic review suggested that no representative model has been constructed using data from large‐sample, multisource, prospective cohorts in China.^12^ At present, the models for the Chinese population are mostly based on small sample, single‐center retrospective research, and extrapolation is limited^13^, ^14^, ^15^, ^16^, ^17^; existing and widely used models are generally based on European and American populations,^18^, ^19^, ^20^, ^21^, ^22^, ^23^ and may not be appropriate for the screening of Asian and Chinese populations, which have unique demographic characteristics.^12^ To fill this gap, our study established a progressive risk prediction model based on the imaging characteristics of pulmonary nodules. The model was constructed using data from the National Lung Cancer Screening (NLCS), a large cohort multicenter prospective cancer screening program. An independent external validation set was created using data from the HMCC (Hospital‐based Medical Checkup Cohort), a cancer screening program composed of two hospital‐based data sources.

In addition, most existing models are based on the whole population or only heavy smokers, and few are based on nonsmoking populations.^12^ Lung cancer in ever‐smokers and nonsmokers shows genetically and statistically different characteristics,^24^, ^25^ but previous separate models based on these two subgroups were insufficient^12^; thus, relevant discussion and evidence were lacking. Therefore, further in‐depth models based on distinct population characteristics were created separately for ever‐smokers and nonsmokers.

## 2 METHODS

### 2.1 Study population

The training set built on data from the NLCS, a cancer screening program initiated in October 2012 aimed at preventing cancer in urban areas. Our study used the data of NLCS enrolled from 2013 to 2018. Data from January 2013 to June 2021 were collected from patients in 12 cities across eight provinces (Beijing, Liaoning, Henan, Hunan, Zhejiang, Anhui, Jiangsu, and Guangxi) who (1) had cancer registration data; (2) had complete vital statistics data (age, sex, etc.); and (3) had a low migration rate and were relatively stable. Eligible participants who provided written informed consent completed a baseline questionnaire that collected information on their exposure to risk factors and evaluated their lung cancer risk using the NLCS scoring system, which introduced in our former research.^6^ Participants assessed as high‐risk were invited to undergo LDCT screening.

Participants were excluded if they met one or more of the following criteria, which were confirmed by the registered residence system of the local community: (1) lung cancer symptoms or cancer diagnosis prior to cohort entry; (2) age outside the age range of 40–74 years; (3) death prior to the cohort entry; or (4) invalid data. For the high‐risk group, patients with invalid data referred to those who did not meet the high‐risk criteria but were classified as high‐risk individuals. For the low‐risk group, patients with invalid data referred to those who were (i) high‐risk individuals misclassified as low‐risk or (ii) labeled as at low‐risk for lung cancer but undertook the free LDCT scan. More details were published in our former research.^6^

Data from individuals with positive nodules detected by LDCT scans were included and used for model construction. According to the NLCS protocol, positive nodules were considered (1) solid or part‐solid nodules larger than 5 mm in diameter; (2) nonsolid nodules larger than 8 mm in diameter; or (3) nodules suspected to be positive on the basis of imaging findings. Only the most representative nodule from each individual with multiple positive nodules was selected for analysis. The most representative nodules were chosen using the following criteria: (1) the nodules were noncalcified; (2) if the degree of calcification of two nodules was the same, the one with a larger mean diameter was chosen.

The validation set was created using data from the HMCC, a hospital‐based physical examination program. Patients who underwent LDCT screening in the Cancer Hospital, Chinese Academy of Medical Sciences, Beijing, China, from January 2017 to December 2017 and the Second People's Hospital of Liaocheng, Shandong, China, from February 2007 to January 2022 were identified. Participants of any age who had at least one suspicious pulmonary nodule were included in the study.

Our study was approved by the ethics committees of the China National Cancer Center/Cancer Hospital, the Chinese Academy of Medical Sciences, and Peking Union Medical College. All participants provided written informed consent. Ethical approval was obtained for all data collection.

### 2.2 Outcome ascertainment

The outcome of this study was the incidence of lung cancer within half a year after LDCT screening. The International Classification of Diseases (10th revision) codes were used for data management, and lung cancer was encoded as C34.

For the training and validation sets, the following information was recorded by the tumor registration system: whether lung cancer was diagnosed, the time of diagnosis, the pathological type, and the clinical stage. All lung cancer cases were based on pathological results and clinical manifestations and issued by professional clinicians, and cross‐referenced between the cancer registry system, local medical insurance databases, and hospital information systems and reviewed by professional clinicians from the Cancer Hospital, Chinese Academy of Medical Sciences, and provincial hospitals. To guarantee the consistency of outcomes, about 1% of all original images were reviewed by professional clinicians. When discrepancies in diagnoses were observed, the records were manually reviewed by at least one thoracic surgeon, one radiologist, and one pathologist from the clinical expert committee.^6^

### 2.3 Data collection

Demographic factors and clinical characteristics were collected using paper‐based and computer‐based forms (epidemiological questionnaire, LDCT report, follow‐up information, pathology report, etc.) in our screening program. Each participant had an identification code for management and traceability. The images of nodules were observed by multirow (64 rows) spiral CT (at least 16 rows); the long diameter and short diameter were measured at the largest section of the nodule with an electronic measuring ruler (self‐contained in the workstation or Picture Archiving and Communication System [PACS]). The report was issued by a senior radiologist with at least 3 years of experience. All the data were saved and analyzed in the National Cancer Prevention and Control Network (NCPCN) at the National Cancer Center (NCC) of China.

The following information was recorded: age, gender, body mass index (BMI), education level (low, defined as primary school or below; medium, defined as intermediate, i.e., junior school to high school; and high, defined as college or above), occupational exposure (harmful working conditions, such as asbestos and dust exposure; yes or no), smoking pack‐years (a pack‐year was defined as 20 cigarettes smoked every day for 1 year), emphysema (yes or no), history of chronic respiratory diseases (yes or no), and family history of lung cancer (yes or no). In addition, the following nodule imaging information was collected: maximum diameter (the longest diameter of the largest section of the nodule) and minimum diameter (the longest diameter perpendicular to the maximum diameter), mean diameter (mean of the maximum and minimum diameters), density (solid, part‐solid, or nonsolid), edge (smooth or spiculated), location in the upper lobe of the lung (yes or no), shape (elliptical or round; a maximum diameter to minimum diameter ratio of ≥1.8 was defined as elliptical, and a ratio of <1.8 was defined as round), pleural involvement (yes or no), and calcification (yes or no). The validation set only collected variables that were eventually incorporated into the models.

### 2.4 Statistical analysis

The statistical analysis was performed with R software, version 4.0.3. Student's *t*‐test was used to compare the quantitative variables, and analysis of variance (ANOVA) or chi‐squared (χ^2^) analyses were used to assess differences between participants who developed incident cancer and those determined to be cancer‐free. Standard mean differences (SMDs) were also used to evaluate differences between groups. Multivariable logistic regression models were developed, and the stepwise regression method was used to select the model with the highest fitting degree according to the value of the Akaike information criterion (AIC). Nonlinear trends of effects were estimated using restricted cubic splines. The beta coefficients, odds ratios (ORs), and 95% confidence intervals (95% CIs) of these were used to discover the associations between covariates and lung cancer risk from the fitted models. The area under the curve (AUC) was used to assess the performance of the models. Calibration performance was measured by plotting the predicted malignancy against the actual outcomes by deciles. Bootstrap resampling conducted 1000 times was used for internal validation. Existing models based on similar factors were compared with our model for the whole population in the validation set. A simulated validation dataset was constructed by bootstrap resampling (1000 times) in the original validation set. The resampled data yielded an incidence rate consistent with that of the training set and were used to simulate the discrimination and calibration in real screening programs.

## 3 RESULTS

### 3.1 Baseline characteristics of the study population

**FIGURE 1:**
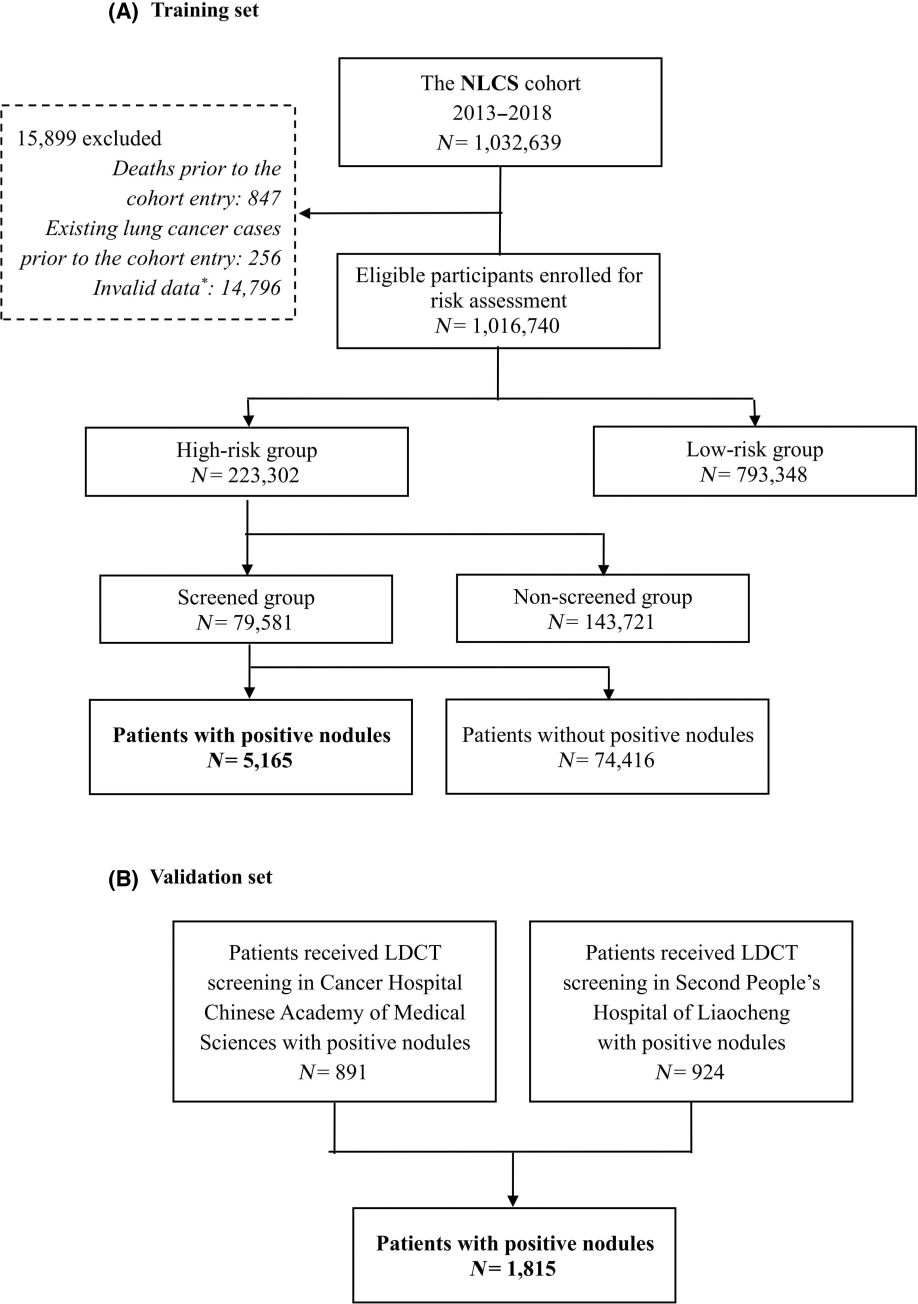
Study profile. * Invalid data included those: (i) who did not meet the high‐risk criteria but were classified as high‐risk individuals; (ii) who were actually high‐risks but were misclassified as low‐risks; (iii) who were low‐risks but undertook the free LDCT scan.

In the NLCS cohort, 1,016,740 participants received risk assessment, of whom 223,302 participants were assessed as high‐risk; 79,581 high‐risk participants underwent LDCT screening, of whom 5165 with at least one suspicious pulmonary nodule were allocated to the training set. The validation set comprised 1815 patients from the Cancer Hospital, Chinese Academy of Medical Sciences, Beijing, and the Second People's Hospital in Liaocheng, Shandong. Figure 1 provides the details of the study profile.

In the training set, 149 (2.9%) participants were diagnosed with lung cancer within half a year, and the FPR was 97.1%. At baseline, the mean age of the training set participants was 58.26 ± 7.66 years. In the validation set, 800 (44.1%) participants were diagnosed with lung cancer within half a year, and the FPR was 55.9%; on the date of LDCT screening, the mean age of the participants was 52.21 ± 11.09 years. The older, underweight participants' nodules had a higher ratio of malignancies. Noncalcified nodules with large diameters, high ratios of nonsolid composition, spiculate edges, locations in the upper lobes, and pleural involvement were also associated with a higher risk of lung cancer in the training set (*p* < 0.05, Table 1).

**TABLE 1:** Baseline characters of the participants.

	Training set					Validation set				
Characters	Overall (*N* = 5165)	Benign (*N* = 5016)	Malignant (*N* = 149)	*P* value	SMD	Overall (*N* = 1815)	Benign (*N* = 1015)	Malignant (*N* = 800)	*P* value	SMD
Age (mean ± SD)	58.26 ± 7.66	58.20 ± 7.68	60.26 ± 6.52	0.001	0.288	52.21 ± 11.09	50.81 ± 11.50	53.98 ± 10.30	<0.001	0.290
Sex				0.775	0.031					
Male	2988 (57.9)	2904 (57.9)	84 (56.4)			774 (42.6)	457 (45.0)	317 (39.6)	<0.001	0.381
Female	2177 (42.1)	2112 (42.1)	65 (43.6)			730 (40.2)	448 (44.1)	282 (35.2)		
Missing	0	0	0			311 (17.1)	110 (10.8)	201 (25.1)		
Education				0.716	0.067					
Low	1206 (23.3)	1170 (23.3)	36 (24.2)							
Medium	3296 (63.8)	3205 (63.9)	91 (61.1)							
High	663 (12.8)	641 (12.8)	22 (14.8)							
Body mass index (kg/m^2^)				0.003	0.292					
<18·5	142 (2.7)	132 (2.6)	10 (6.7)							
18·5–24	2616 (50.6)	2540 (50.6)	76 (51.0)							
24–28	1965 (38.0)	1907 (38.0)	58 (38.9)							
≥28	442 (8.6)	437 (8.7)	5 (3.4)							
Frequent exercise				0.302	0.096					
No	3742 (72.4)	3628 (72.3)	114 (76.5)							
Yes	1423 (27.6)	1388 (27.7)	35 (23.5)							
Passive smoking year				0.935	0.054					
No	938 (18.2)	910 (18.1)	28 (18.8)							
0–19	593 (11.5)	577 (11.5)	16 (10.7)							
20–39	2615 (50.6)	2542 (50.7)	73 (49.0)							
≥40	1019 (19.7)	987 (19.7)	32 (21.5)							
Smoking status				0.979	0.009				0.576	0.050
Never‐smoker	1780 (34.5)	1728 (34.4)	52 (34.9)			1282 (70.6)	709 (69.9)	573 (71.6)		
Ever‐smoker	3385 (65.5)	3288 (65.6)	97 (65.1)			521 (28.7)	298 (29.4)	223 (27.9)		
Missing	0	0	0			12 (0.7)	8 (0.8)	4 (0.5)		
Family history of lung cancer				0.105	0.142					
No	2574 (49.8)	2510 (50.0)	64 (43.0)							
Yes	2591 (50.2)	2506 (50.0)	85 (57.0)							
Chronic respiratory diseases				0.342	0.088					
No	1515 (29.3)	1477 (29.4)	38 (25.5)							
Yes	3650 (70.7)	3539 (70.6)	111 (74.5)							
Emphysema				0.957	0.015					
No	4600 (89.1)	4468 (89.1)	132 (88.6)							
Yes	565 (10.9)	548 (10.9)	17 (11.4)							
Maximum diameter(mm)	9.35 ± 7.06	9.03 ± 6.45	20.26 ± 14.29	<0.001	1.013	12.95 ± 9.92	11.73 ± 9.91	14.52 ± 9.71	<0.001	0.285
Missing	0	0	0			39 (2.1)	16 (1.6)	23 (2.9)		
Minimum diameter(mm)	7.19 ± 5.23	6.94 ± 4.80	15.45 ± 10.07	<0.001	1.079	10.12 ± 7.80	9.22 ± 7.78	11.27 ± 7.67	<0.001	0.265
Missing	0	0	0			33 (1.8)	20 (2.0)	13 (1.6)		
Location				0.001	0.277					
Not upper lobe	3215 (62.2)	3142 (62.6)	73 (49.0)							
Upper lobe	1950 (37.8)	1874 (37.4)	76 (51.0)							
Density				<0.001	0.464				<0.001	0.713
Solid	3878 (75.1)	3797 (75.7)	81 (54.4)			907 (50.0)	642 (63.3)	265 (33.1)		
Part‐solid	785 (15.2)	740 (14.8)	45 (30.2)			392 (21.6)	112 (11.0)	280 (35.0)		
Nonsolid	502 (9.7)	479 (9.5)	23 (15.4)			434 (23.9)	220 (21.7)	214 (26.8)		
Missing	0	0	0			82 (4.5)	41 (4.0)	41 (5.1)		
Calcification				0.002	0.334				0.001	0.184
No	4550 (88.1)	4406 (87.8)	144 (96.6)			1732 (95.4)	959 (94.5)	773 (96.6)		
Yes	615 (11.9)	610 (12.2)	5 (3.4)			57 (3.1)	45 (4.4)	12 (1.5)		
Missing	0	0	0			26 (1.4)	11 (1.1)	15 (1.9)		
Pleural involvement				<0.001	0.603				<0.001	0.439
No	4244 (82.2)	4160 (82.9)	84 (56.4)			1323 (72.9)	826 (81.4)	497 (62.1)		
Yes	921 (17.8)	856 (17.1)	65 (43.6)			465 (25.6)	177 (17.4)	288 (36.0)		
Missing	0	0	0			27 (1.5)	12 (1.2)	15 (1.9)		
Edge				<0.001	0.314				0.009	0.146
Smooth	3433 (66.5)	3356 (66.9)	77 (51.7)			473 (26.1)	290 (28.6)	183 (22.9)		
Spiculated	1732 (33.5)	1660 (33.1)	72 (48.3)			1315 (72.5)	714 (70.3)	601 (75.1)		
Missing	0	0	0			27 (1.5)	11 (1.1)	16 (2.0)		
Shape				0.565	0.064					
Round	4772 (92.4)	4632 (92.3)	140 (94.0)							
Ellipse	393 (7.6)	384 (7.7)	9 (6.0)							

*Note*: Data were presented as mean ± SD (standard derivation) or *n* (%) unless otherwise specified.

The baseline characteristics of ever‐smokers and never‐smokers are displayed in Tables S1 and S2. A total of 3385 (65.5%) ever‐smokers and 1780 (34.5%) never‐smokers were used for the model construction in the subgroups. The incidence of lung cancer is 2.9% for both ever‐smokers (97/3385) and nonsmokers (52/1780), with a FPR of 97.1%. Data from 521 (28.9%) ever‐smokers and 1282 (71.1%) never‐smokers were used for validation.

### 3.2 Model development and evaluation

**FIGURE 2:**
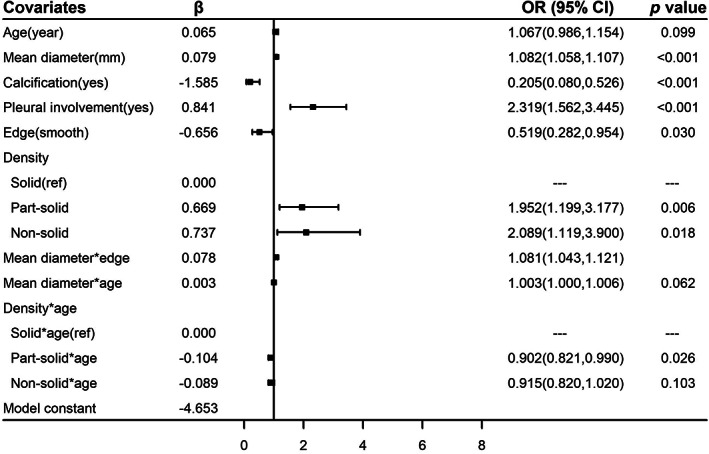
Effectiveness of covariates in the final model in whole population. *Age in our models was performed with the following calculation: (Age‐60). 61 years old and older participants were defined as 60 years old due to the nonlinear association of age and lung cancer risk.

Six covariates (the age of the participants and the mean diameter, calcification, pleural involvement, edge, and density of the nodules) and their interaction effects associated with lung cancer were entered into the prediction model through multivariate logistic regression. The age of the participants (OR = 1.067, 95% CI: 0.986–1.154) and the mean diameter (OR = 1.082, 95% CI: 1.058–1.107), calcification (OR = 0.205, 95% CI: 0.080–0.526), pleural involvement (OR = 2.319, 95% CI: 1.562–3.445), smooth edges (OR = 0.519, 95% CI: 0.282–0.954), and density (part‐solid vs. solid: OR = 1.952, 95% CI: 1.199–3.177; nonsolid vs. solid: OR = 2.089, 95% CI: 1.119–3.900) of the nodules were related to lung cancer risk. The model for the whole population and the relative risk of the covariates are shown in Figure 2.

Significant interaction effects were observed between age and density (part‐solid × age vs. solid × age: OR = 0.902, 95% CI: 0.821–0.990; nonsolid × age vs. solid × age: OR = 0.915, 95% CI: 0.820–1.020), age and mean diameter (OR = 1.003, 95% CI: 1.000–1.006), and edge and mean diameter (OR = 1.081, 95% CI: 1.043–1.121); these were entered into the model (Figure 2). The correlation between age and incidence was nonlinear because of the result of restricted cubic spline; in patients over 60 years old, lung cancer risk did not change much with increasing age (Figure S1). Thus, in our models, the risk of participants over 60 years old was calculated as a constant.

**FIGURE 3:**
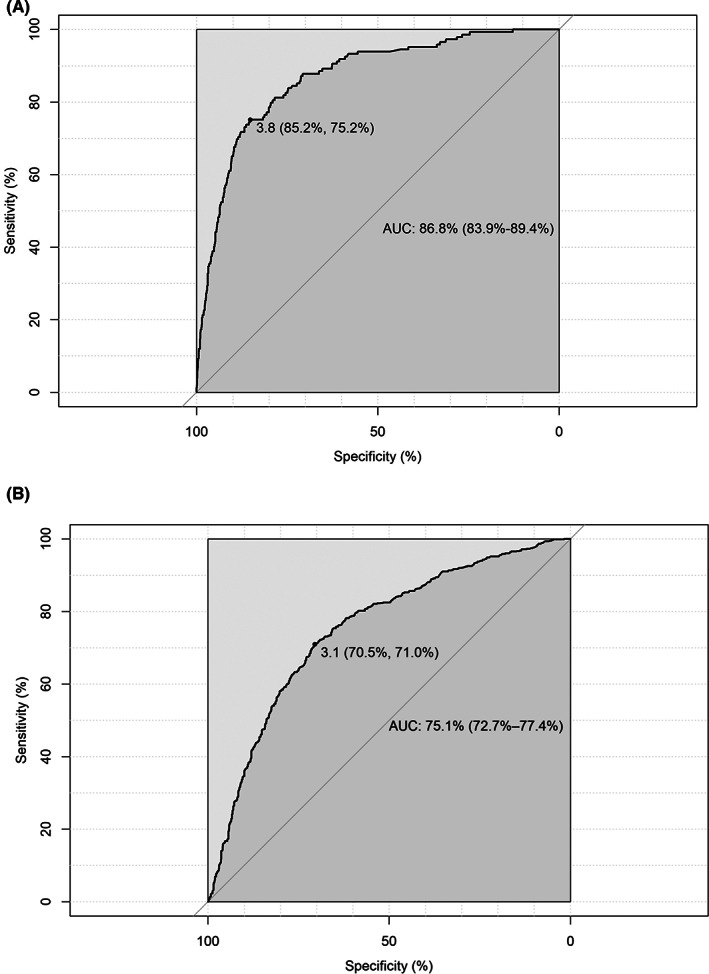
Discrimination of the final model in whole population. (A) internal validation; (B) external validation.

**FIGURE 4:**
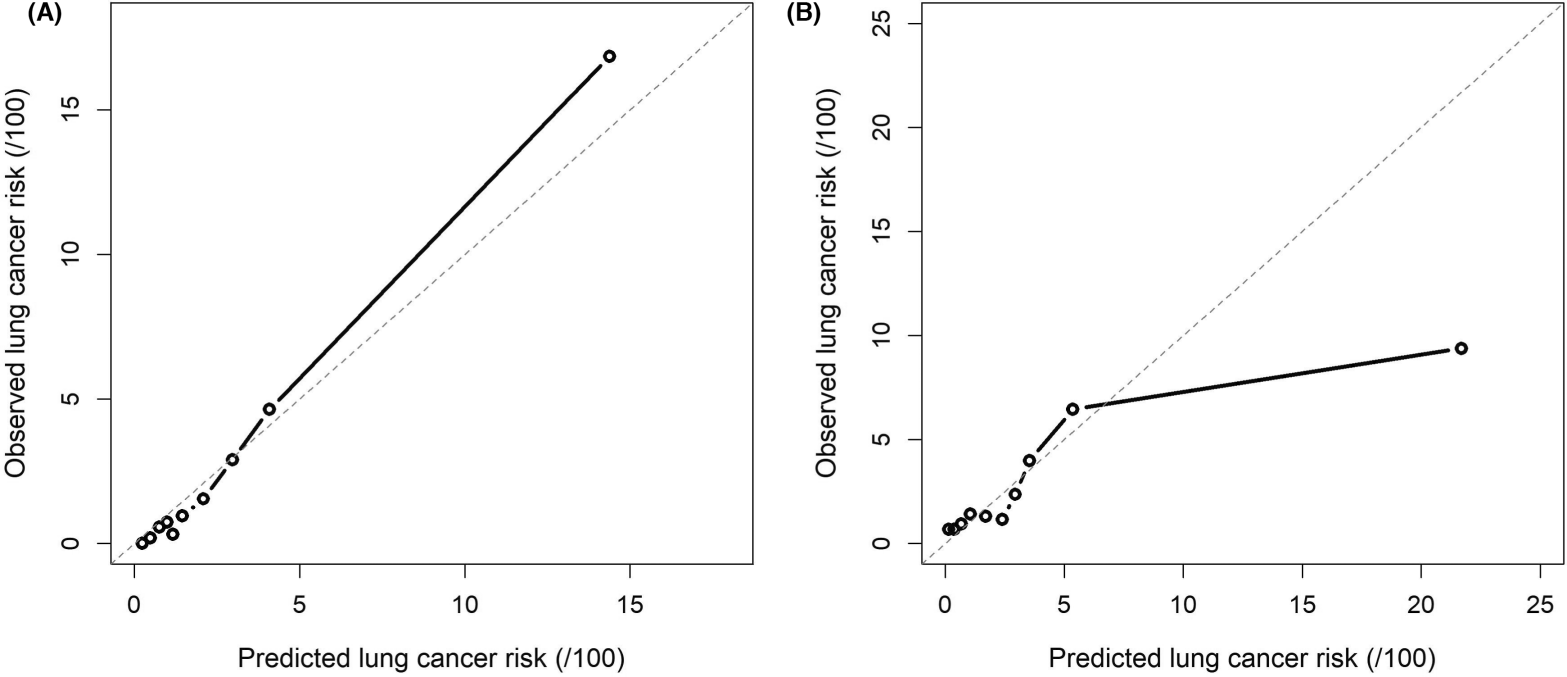
Calibration of the final model in whole population. (A) internal validation; (B) external validation.

The model in whole population showed good discriminative capacity in internal validation (AUC = 0.868, 95% CI: 0.839–0.894, Figure 3A) and calibration (Figure 4A) by bootstrap resampling 1000 times. The sensitivity and specificity were 85.2% and 75.2%, respectively, with a 3.9% risk threshold, indicating that our model could reduce the 72.9% FPR in the screening cohort. Online calculator was provided at http://cancerrc.ncsis.org.cn/#/lungCancer for the calculation of lung cancer probability.

**FIGURE 5:**
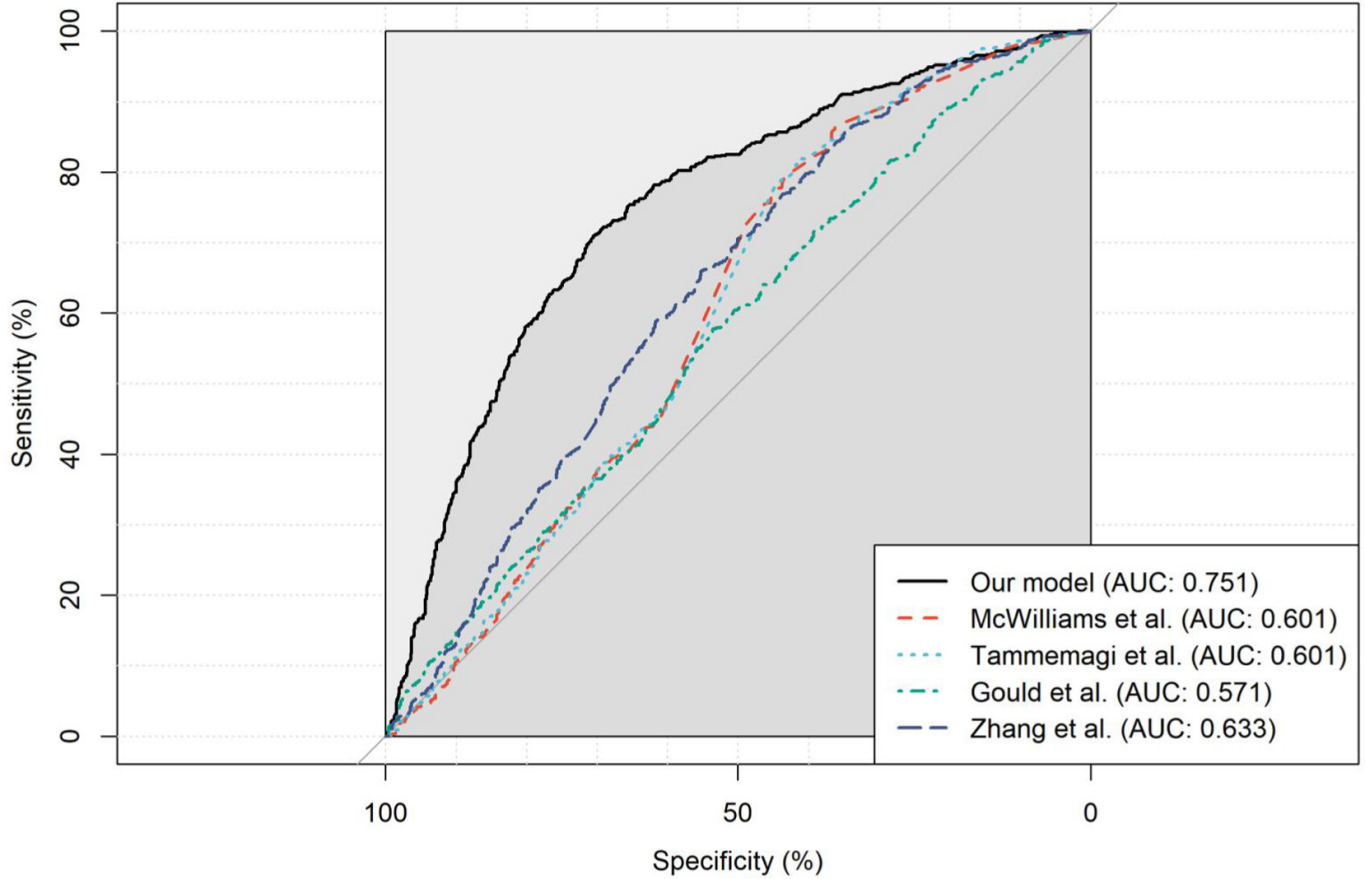
Comparison of discrimination of different models in the validation set.

Compared with existing models based on similar factors (Zhang et al.^17^; Gould et al.^20^; PanCan 1A, McWilliams et al.^21^; and Tammemagi et al.^22^), our model obtained a higher AUC in the Chinese population (Figure 5); the consideration of the nonlinear trend of age and the interaction effects also improved the discrimination (Figure S2).

In the validation set, our model in whole population also showed excellent discrimination (AUC = 0.751, 95% CI: 0.727–0.774, Figure 3B). When the threshold was 3.1%, the sensitivity and specificity reached 70.5% and 71.0%, respectively. Compared with other existing models, our model fit better with actual lung cancer incidence, demonstrated by lower absolute errors (observed minus predicted probabilities) in the resampled validation set (Table S3). In the simulated validation set, the AUC was 0.752 (95% CI: 0.709–0.788) with 70.5% sensitivity and 70.9% specificity at a 3.1% risk threshold, which may reduce the 68.8% FPR in LDCT screening. The predicted and observed outcomes matched well in most cases in the validation set (Figure 4B).

The models based on smokers (AUC = 0.732, 95% CI: 0.686–0.778) and nonsmokers (AUC = 0.740, 95% CI: 0.712–0.767) also demonstrated excellent discrimination in the validation set (Figure S3). Good calibration was still observed in most cases in the resampled validation sets (Figure S4). The details of models are displayed in Figure S5. The included variables of the two models, however, did not differ significantly.

## 4 DISCUSSION

Our study constructed models based on imaging and epidemiological factors that aimed to predict the risk of lung cancer. High‐quality evidence obtained from a prospective, multicenter cohort study in China was used for model construction. We applied a selection strategy that focused on the most suspicious cases and optimized the existing judgment criteria for positive screening results. Validation was performed in an independent dataset, which comprised data collected from different cities. The results of internal and external validation by bootstrap resampling showed that our models can reduce the FPR and overdiagnosis in lung cancer screening programs. Compared with other models based on similar factors, our model in whole population performed better in the validation set. With the support of independent external validation, our study has pioneering significance for Chinese lung cancer screening.

Lung cancer in China and Asia is unique in terms of epidemiological characteristics; for example, 30% of lung cancer cases in China are not attributable to smoking, a proportion much higher than that in the American population (20%).^22^, ^26^ Existing models, which are generally based on American and European populations, may be inefficient when applied to Asian populations because of the differences in population characteristics; the validation of these models in our dataset supports this. Models constructed on Chinese and Asian populations may have bias originating from small sample sizes, retrospective study designs, and single data sources.^12^ Models based on deep learning algorithms have shown good discrimination,^27^, ^28^, ^29^ but the demand for large numbers of covariates may limit their clinical application. Our research provides a viable solution to these problems.

Our study considered widely recognized lung cancer risk factors,^12^ including traditional epidemiological factors and imaging features of nodules. Therefore, our models reflect the epidemiological and imaging risk factors in the Chinese population to the greatest extent. Our ultimate models were mainly constructed on imaging factors, and most of the macrolevel epidemiological factors were not statistically significant. Previous research revealed that the personal statements of patients may have great bias^30^; however, our models use only age as an effective epidemiological predictor, which means that most of the possible random measurement bias from self‐reporting is avoided, and a higher application value in actual screening can be expected. Our models primarily depend on imaging variables, which makes them easier to apply in practical LDCT screening protocols.

In addition, in former Chinese researches, interaction effects were rarely mentioned.^12^ To our knowledge, our research is the first to consider interaction effects in the Chinese population, and it achieved superior performance compared with previous models. Our findings reveal that the mechanisms of action between different factors are not independent and should not be ignored.

However, smoking was not considered a significant predictor, which was contrary to common sense, but could be explained by the unique population characteristics in Asia and China. In the researches of Bin Zheng et al.,^13^ Sungmin Zo et al.,^31^ and Xiaobo Chen et al.,^32^ smoking status was not an independent risk factor of lung cancer in prediction models for Asian populations. Furthermore, the differences between models for ever‐smokers and nonsmokers were not obvious; similar risk factors and effects were observed in the two models. Therefore, we believe that the degree of malignancy depends more on the imaging characteristics of the nodules than on smoking status in the Asian population.

Our previous research has confirmed that simultaneous screening for smokers and nonsmokers based on the same risk of lung cancer is currently the most effective screening strategy in the Chinese population.^24^ The study, however, did not take into account the difference in FPR and ensuing management challenges in positive screening results. The effectiveness of screening will be relatively low if suspicious nodules in nonsmokers have a higher FPR or are more challenging to accurately assess their risk of lung cancer. This means that this population will undergo more unnecessary diagnoses and treatments. This study indicates that the FPR is equal between smokers and nonsmokers, and the models' discrimination and calibration are also comparable. This suggests that the cost of unnecessary diagnosis and treatment is similar for both groups. The results of this study fill the evidence gap and provide strong evidence to support lung cancer screening in nonsmokers in China.

Our study has several limitations. Our research used data from one‐off LDCT screening^6^; therefore, variables that changed over time, some of which may associated with lung cancer risk,^33^ could not be assessed. Biomarkers and environmental factors were not considered for practical and economic reasons. In addition, our models were constructed for large‐scale lung cancer screening; however, our validation set used retrospective clinical data. Although the validation set was also derived from individuals with suspicious nodules detected by LDCT, it was not established based on random sampling or a strict definition of high‐risk groups. Compared to real‐world screening, it was difficult to avoid the bias brought by retrospective study design. As a result, the data exhibits differences in population characteristics, which are reflected in differences in risk factors and a higher risk of lung cancer in this study. Due to differences in population characteristics, it may lead to lower discrimination and calibration of models during external validation. To evaluate the models' calibration for actual screening, simulated datasets with incidence rates consistent with those of the training set must be constructed by bootstrap resampling; however, more examinations in future screening programs are required.

In summary, our research and its application prospects are extensive. In the training and validation sets, our model in whole population achieved good discrimination and calibration, indicating that it performs well and is suitable for large‐scale application. In the next large‐scale LDCT screening in China, it could guide lung cancer screening, nodule stratification, and further follow‐up management, ultimately reducing the disease burden of lung cancer.

## AUTHOR CONTRIBUTIONS

**Zheng Wu:** Conceptualization (equal); data curation (lead); formal analysis (lead); methodology (equal); validation (equal); visualization (supporting); writing – original draft (lead); writing – review and editing (equal). **Fengwei Tan:** Conceptualization (equal); funding acquisition (equal); methodology (equal); project administration (equal); resources (equal); supervision (equal); writing – review and editing (supporting). **Yaozeng Xie:** Data curation (equal); investigation (equal); resources (equal); validation (equal); writing – review and editing (equal). **Wei Tang:** Conceptualization (equal); data curation (equal); project administration (equal); supervision (equal); writing – review and editing (equal). **Fei Wang:** Conceptualization (equal); formal analysis (supporting); funding acquisition (supporting); resources (supporting); validation (supporting); writing – original draft (supporting); writing – review and editing (equal). **Yongjie Xu:** Project administration (supporting); resources (supporting); writing – review and editing (equal). **Wei Cao:** Data curation (supporting); methodology (supporting); project administration (supporting); resources (supporting); writing – review and editing (equal). **Chao Qin:** Methodology (supporting); project administration (supporting); software (lead); writing – review and editing (equal). **Xuesi Dong:** Conceptualization (supporting); funding acquisition (supporting); writing – review and editing (equal). **Yadi Zheng:** Data curation (supporting); writing – review and editing (equal). **Zilin Luo:** Data curation (supporting); writing – review and editing (equal). **Chenran Wang:** Data curation (supporting); writing – review and editing (equal). **Liang Zhao:** Data curation (supporting); project administration (supporting); software (supporting); writing – review and editing (supporting). **Changfa Xia:** Formal analysis (supporting); writing – review and editing (supporting). **Jiang Li:** Formal analysis (supporting); writing – review and editing (supporting). **Renda Li:** Resources (supporting); writing – review and editing (supporting). **Feiyue Feng:** Resources (supporting); writing – review and editing (supporting). **Jibin Li:** Formal analysis (supporting); writing – review and editing (supporting). **Jiansong Ren:** Formal analysis (supporting); writing – review and editing (supporting). **Ju‐Fang Shi:** Formal analysis (supporting); writing – review and editing (supporting). **Hong Cui:** Resources (supporting); writing – review and editing (supporting). **Sipeng Shen:** Formal analysis (supporting); methodology (supporting); writing – review and editing (supporting). **Ning Wu:** Methodology (supporting); project administration (supporting); resources (supporting); supervision (supporting); writing – review and editing (equal). **Wanqing Chen:** Project administration (supporting); resources (supporting); supervision (supporting); writing – review and editing (equal). **Ni Li:** Conceptualization (equal); data curation (supporting); formal analysis (supporting); funding acquisition (lead); investigation (supporting); methodology (supporting); project administration (lead); resources (lead); supervision (lead); visualization (supporting); writing – review and editing (equal). **Jie He:** Conceptualization (supporting); data curation (supporting); funding acquisition (supporting); investigation (supporting); project administration (equal); resources (equal); supervision (equal); writing – review and editing (equal).

## FUNDING INFORMATION

This study was funded by grants from the Ministry of Finance and National Health Commission of the People's Republic of China, National Key Research and Development Program of China (grant number 2021YFC2500900); National Natural Science Foundation of China (grant numbers 82204143 and 82273722); Special Research Fund for Central Universities, Peking Union Medical College (grant number 3332022031); the nonprofit Central Research Institute Fund of the Chinese Academy of Medical Sciences (grant numbers 2019PT320027 and 2020PT330001); the Chinese Academy of Medical Sciences Innovation Fund for Medical Science (grant numbers 2019‐I2M‐2‐002, 2021‐I2M‐1‐011, and 2021‐I2M‐1‐067); the Sanming Project of Medicine in Shenzhen (grant number SZSM201911015); the Beijing Science and Technology Project (grant number Z181100001718212).

## CONFLICT OF INTEREST STATEMENT

The authors declare that they have no competing interests.

## ETHICS STATEMENT

The studies involving human participants were reviewed and approved by the ethic committee of the Chinese Academy of Medical Sciences and Peking Union Medical College. The patients/participants provided their written informed consent to participate in this study.

## Supporting information


Figures S1–S5

Tables S1–S3

## Data Availability

The datasets for this manuscript are not publicly available because all our data are under regulation of the National Cancer Center of China. Requests to access the datasets should be directed to JH, prof.jiehe@gmail.com.
