# Supplementary material for: A strategy to reduce the false‐positive rate after low‐dose computed tomography in lung cancer screening: A multicenter prospective cohort study
Source: Cancer Med. 2023 May 18;12(13):14781–93. doi: 10.1002/cam4.6106 (PMC10358224; doi:10.1002/cam4.6106)
Supplement: Supplementary file 1 — Figures S1–S5 [file CAM4-12-14781-s001.docx]

**
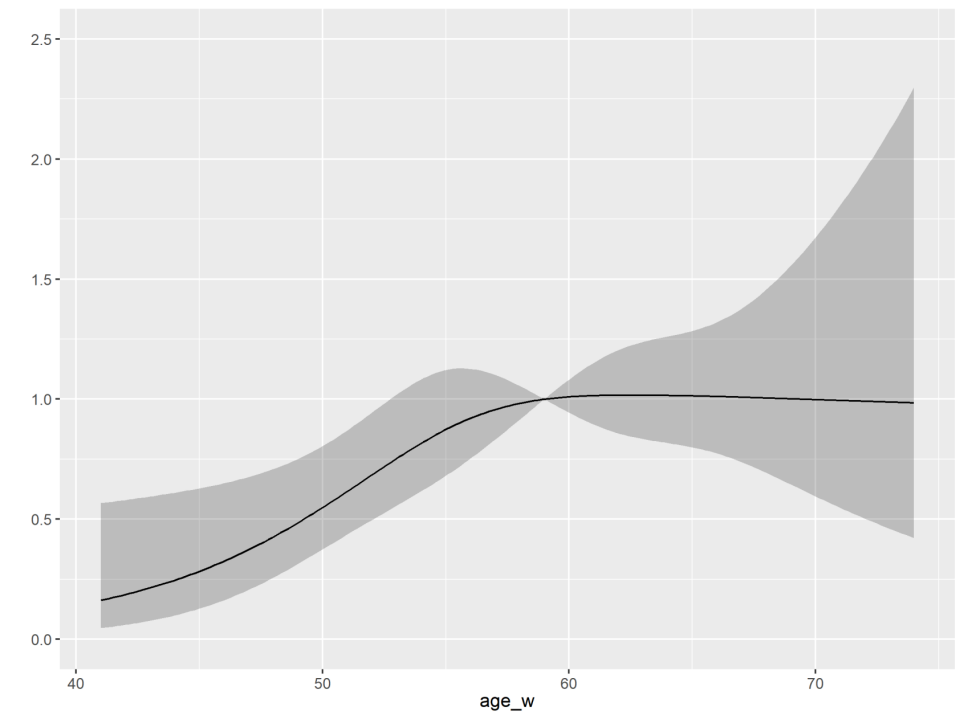
**

**Figure S1 The non-linear association between age and the risk of lung cancer.**

**
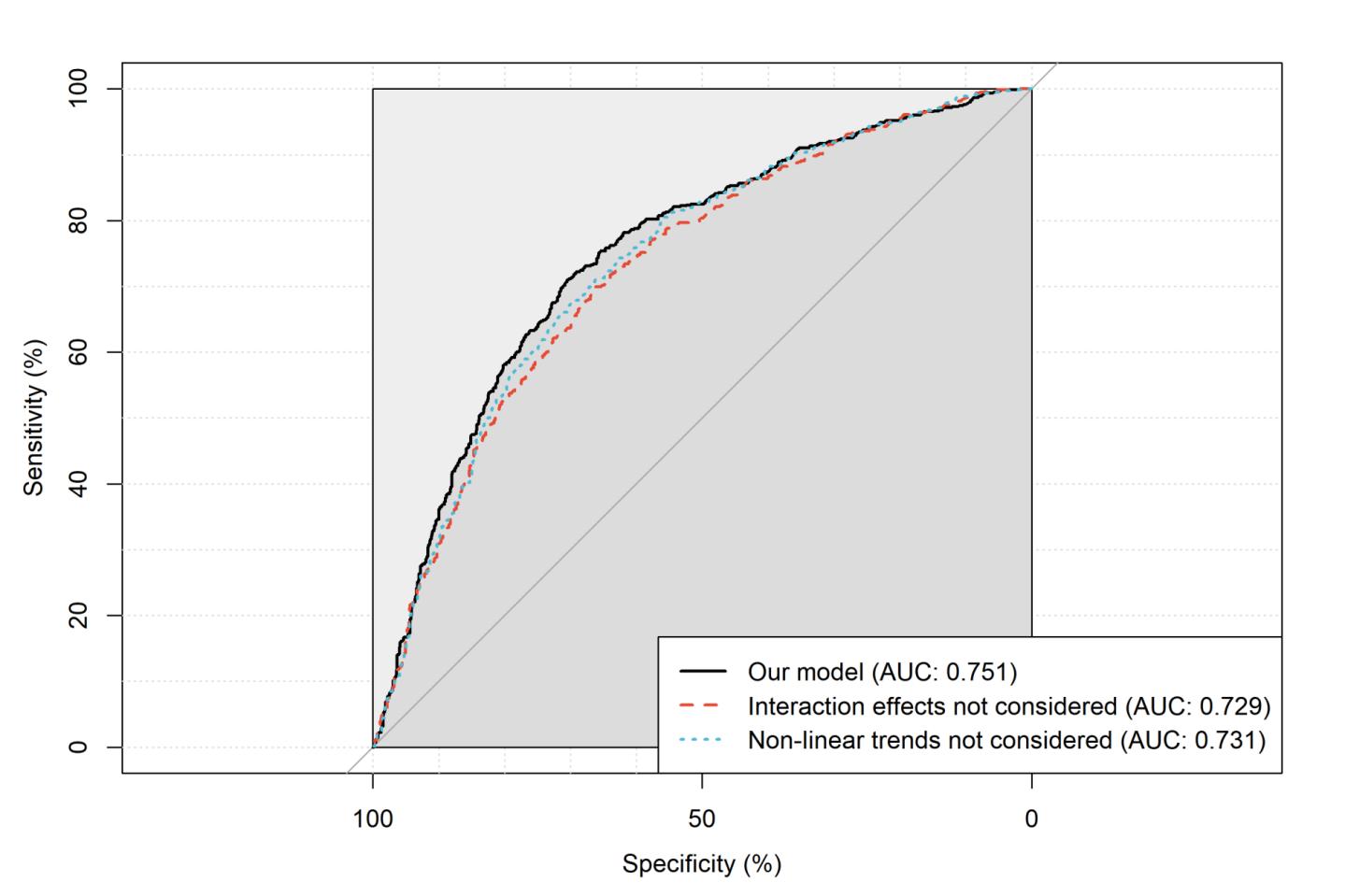
**

**Figure S2 The impact of interaction effects and non-linear effects on the discrimination of the model based on the whole population.**

**
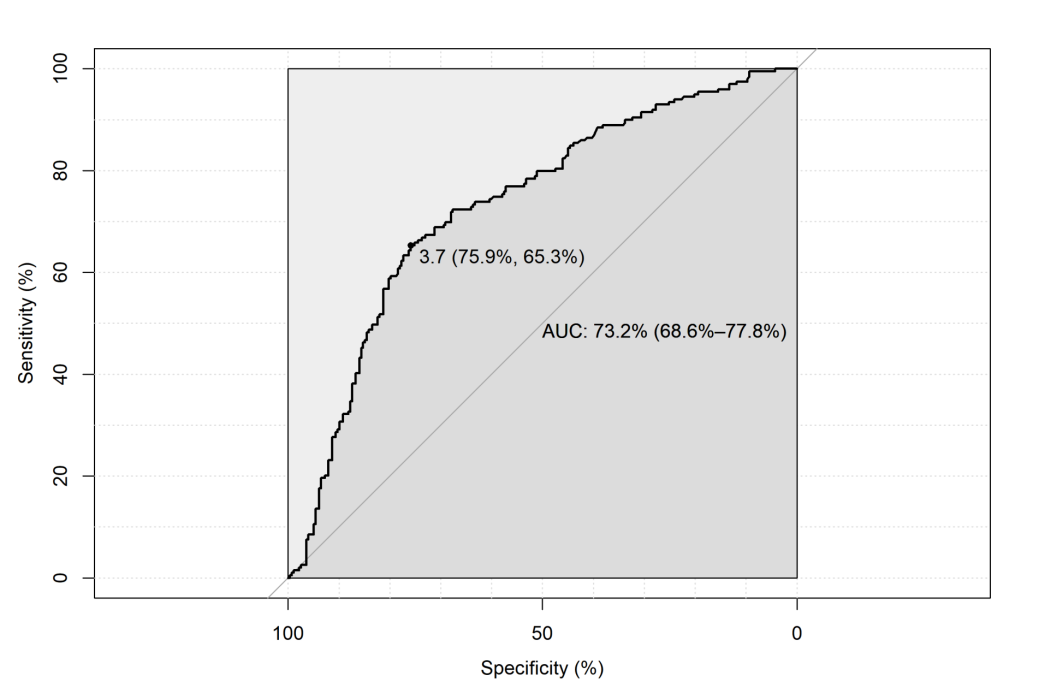

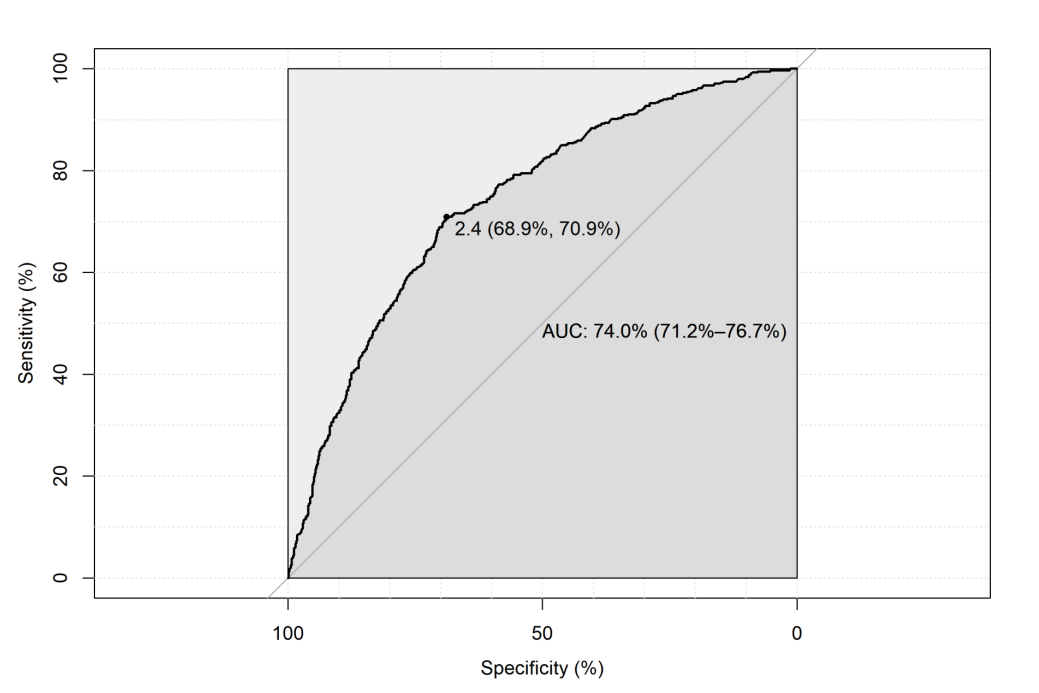
**

B)

A)

**Figure S3 Discrimination of models developed separately for smokers and non-smokers in external validation. A) model for ever-smokers; B) model for never-smokers.**


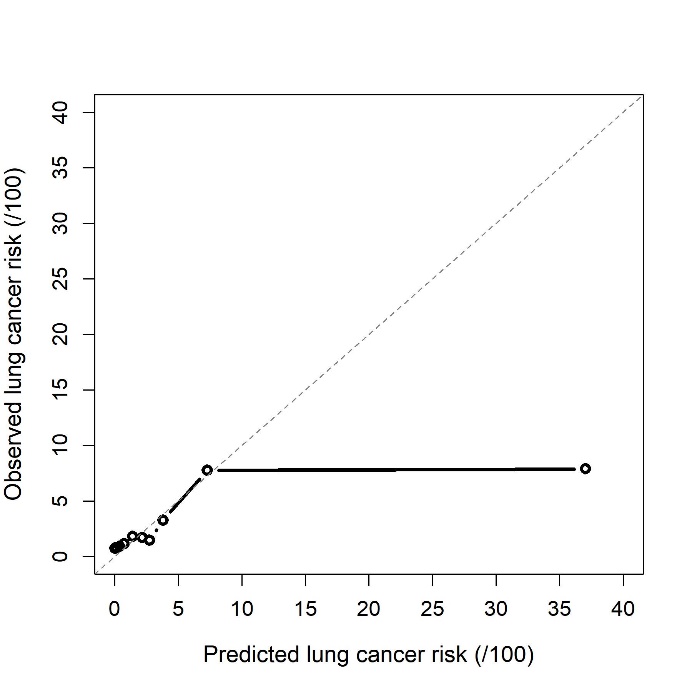

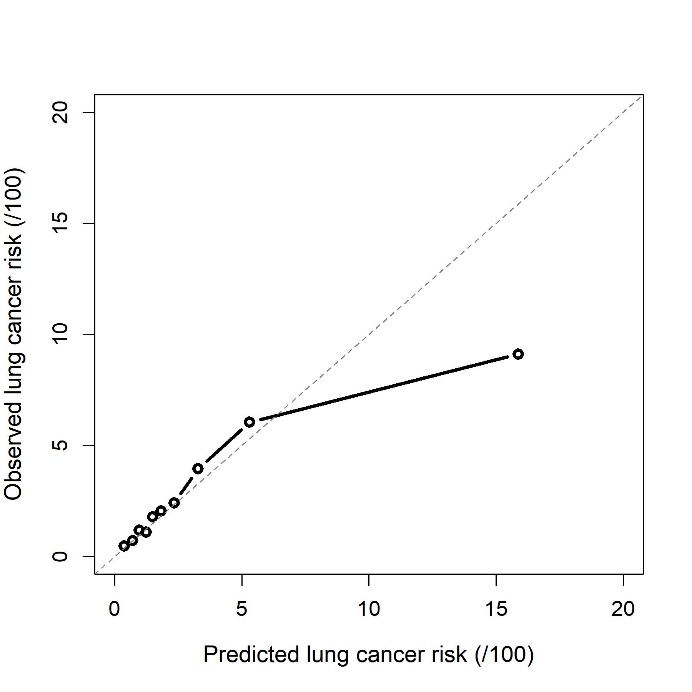


A)

B)

**FigureS4 Calibration of models developed separately for smokers and non-smokers in external validation. A) model for ever-smokers; B) model for never-smokers.**

**
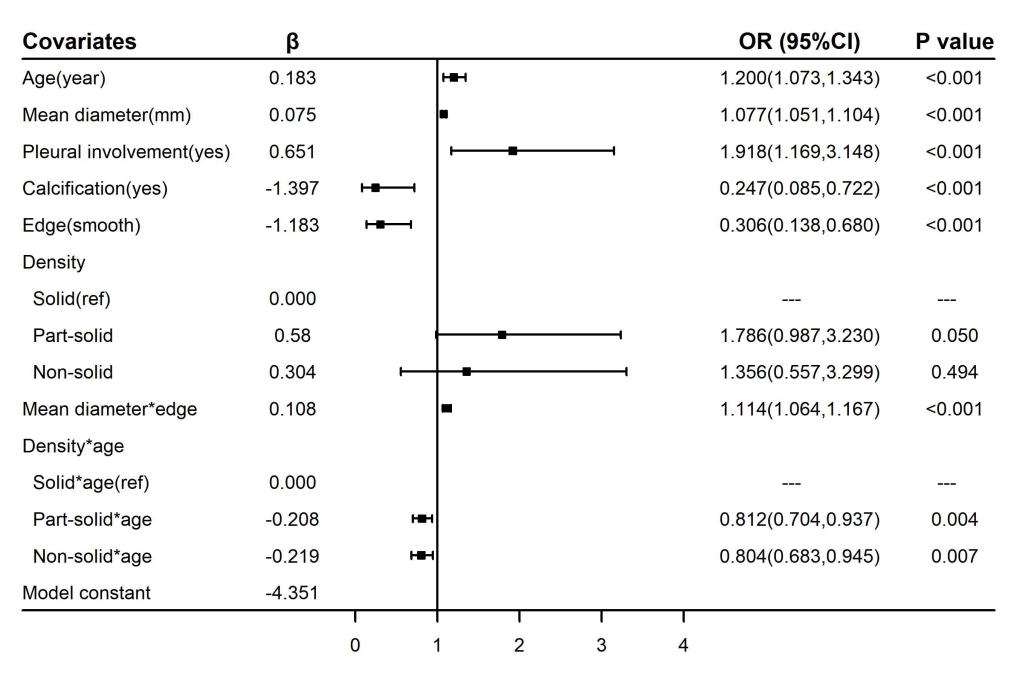

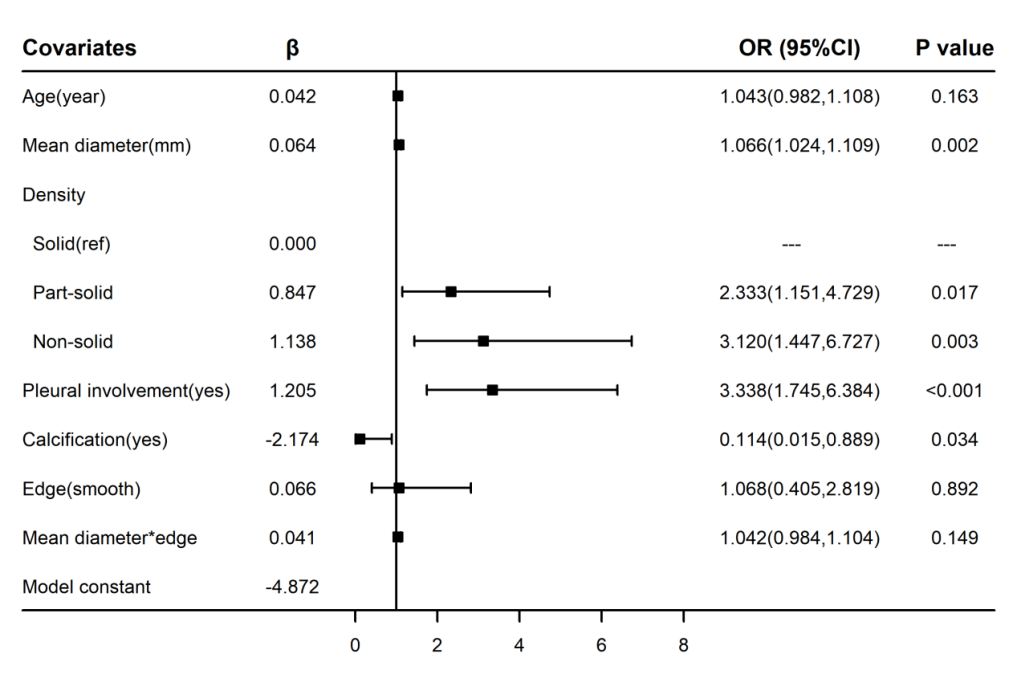
**

B)

A)

**Figure S5 The variable effects of models developed separately for smokers and non-smokers. A) model for ever-smokers; B) model for never-smokers.**

*****Age in our models was performed with the following calculation: (Age-60). 61 years old and older participants were defined as 60 years old due to the non-linear association of age and lung cancer risk.
