# Supplementary material for: A strategy to reduce the false‐positive rate after low‐dose computed tomography in lung cancer screening: A multicenter prospective cohort study
Source: Cancer Med. 2023 May 18;12(13):14781–93. doi: 10.1002/cam4.6106 (PMC10358224; doi:10.1002/cam4.6106)
Supplement: Supplementary file 2 — Tables S1–S3 [file CAM4-12-14781-s002.docx]

**Table S1 Baseline characters of the ever-smokers.**

| **Characters** | **Training set** | | | | | **Validation set** | | | | |
| --- | --- | --- | --- | --- | --- | --- | --- | --- | --- | --- |
|  | **Overall** | **Benign** | **Malignant** | ***P* value** | **SMD** | **Overall** | **Benign** | **Malignant** | ***P* value** | **SMD** |
|  | **(N=3385)** | **(N=3288)** | **(N=97)** |  |  | **(N=521)** | **(N=298)** | **(N=223)** |  |  |
| **Age (mean±SD)** | 58.80± 7.52 | 58.73± 7.54 | 61.18± 6.41 | 0.002 | 0.350 | 54.89±10.49 | 53.08±10.70 | 57.30±9.70 | <0.001 | 0.412 |
| **Sex** |  |  |  | 0.719 | 0.052 |  |  |  | <0.001 | 0.462 |
| Male | 2988 (88.3) | 2904 (88.3) | 84 (86.6) |  |  | 22 (4.2) | 13 (4.4) | 9 (4.0) |  |  |
| Female | 397 (11.7) | 384 (11.7) | 13 (13.4) |  |  | 420 (80.6) | 261 (87.6) | 159 (71.3) |  |  |
| Missing | 0 | 0 | 0 |  |  | 79 (15.2) | 24 (8.1) | 55 (24.7) |  |  |
| **Education** |  |  |  | 0.583 | 0.104 |  |  |  |  |  |
| Low | 744 (22.0) | 721 (21.9) | 23 (23.7) |  |  |  |  |  |  |  |
| Medium | 2213 (65.4) | 2154 (65.5) | 59 (60.8) |  |  |  |  |  |  |  |
| High | 428 (12.6) | 413 (12.6) | 15 (15.5) |  |  |  |  |  |  |  |
| **Body mass index** (kg/m^2^) |  |  |  | 0.013 | 0.292 |  |  |  |  |  |
| <18·5 | 88 (2.6) | 81 (2.5) | 7 (7.2) |  |  |  |  |  |  |  |
| 18·5-24 | 1672 (49.4) | 1624 (49.4) | 48 (49.5) |  |  |  |  |  |  |  |
| 24-28 | 1324 (39.1) | 1286 (39.1) | 38 (39.2) |  |  |  |  |  |  |  |
| ≥28 | 301 (8.9) | 297 (9.0) | 4 (4.1) |  |  |  |  |  |  |  |
| **Frequent exercise** |  |  |  | 0.135 | 0.174 |  |  |  |  |  |
| No | 2443 (72.2) | 2366 (72.0) | 77 (79.4) |  |  |  |  |  |  |  |
| Yes | 942 (27.8) | 922 (28.0) | 20 (20.6) |  |  |  |  |  |  |  |
| **Passive smoking year** |  |  |  | 0.942 | 0.064 |  |  |  |  |  |
| No | 704 (20.8) | 685 (20.8) | 19 (19.6) |  |  |  |  |  |  |  |
| 0-19 | 373 (11.0) | 363 (11.0) | 10 (10.3) |  |  |  |  |  |  |  |
| 20-39 | 1587 (46.9) | 1542 (46.9) | 45 (46.4) |  |  |  |  |  |  |  |
| ≥40 | 721 (21.3) | 698 (21.2) | 23 (23.7) |  |  |  |  |  |  |  |
| **Family history of lung cancer** |  |  |  | 0.222 | 0.136 |  |  |  |  |  |
| No | 1932 (57.1) | 1883 (57.3) | 49 (50.5) |  |  |  |  |  |  |  |
| Yes | 1453 (42.9) | 1405 (42.7) | 48 (49.5) |  |  |  |  |  |  |  |
| **Chronic respiratory diseases** |  |  |  | 0.133 | 0.169 |  |  |  |  |  |
| No | 1420 (41.9) | 1387 (42.2) | 33 (34.0) |  |  |  |  |  |  |  |
| Yes | 1965 (58.1) | 1901 (57.8) | 64 (66.0) |  |  |  |  |  |  |  |
| **Emphysema** |  |  |  | 0.973 | 0.021 |  |  |  |  |  |
| No | 3015 (89.1) | 2928 (89.1) | 87 (89.7) |  |  |  |  |  |  |  |
| Yes | 370 (10.9) | 360 (10.9) | 10 (10.3) |  |  |  |  |  |  |  |
| **Maximum diameter(mm)** | 9.45±7.33 | 9.07±6.51 | 22.51±16.32 | <0.001 | 1.081 | 15.58±11.99 | 13.53±11.57 | 18.37±12.01 | <0.001 | 0.410 |
| Missing | 0 | 0 | 0 |  |  | 20 (3.8) | 9 (3.0) | 11 (4.7) |  |  |
| **Minimum diameter(mm)** | 7.20±5.42 | 6.92±4.86 | 16.91±11.38 | <0.001 | 1.142 | 12.16±9.40 | 10.64±8.73 | 14.20±9.89 | <0.001 | 0.381 |
| Missing | 0 | 0 | 0 |  |  | 17 (3.3) | 9 (3.0) | 8 (3.4) |  |  |
| **Location** |  |  |  | 0.016 | 0.255 |  |  |  |  |  |
| Not upper lobe | 2122 (62.7) | 2073 (63.0) | 49 (50.5) |  |  |  |  |  |  |  |
| Upper lobe | 1263 (37.3) | 1215 (37.0) | 48 (49.5) |  |  |  |  |  |  |  |
| **Density** |  |  |  | <0.001 | 0.438 |  |  |  | <0.001 | 0.468 |
| Solid | 2629 (77.7) | 2572 (78.2) | 57 (58.8) |  |  | 320 (61.4) | 207 (69.5) | 113 (50.7) |  |  |
| Part-solid | 492 (14.5) | 463 (14.1) | 29 (29.9) |  |  | 82 (15.7) | 27 (9.1) | 55 (24.7) |  |  |
| Nonsolid | 264 (7.8) | 253 (7.7) | 11 (11.3) |  |  | 81 (15.5) | 44 (14.8) | 37 (16.6) |  |  |
| Missing | 0 | 0 | 0 |  |  | 38 (7.3) | 20 (6.7) | 18 (8.1) |  |  |
| **Calcification** |  |  |  | 0.029 | 0.289 |  |  |  | 0.097 | 0.198 |
| No | 2990 (88.3) | 2897 (88.1) | 93 (95.9) |  |  | 490 (94.0) | 275 (92.3) | 215 (96.4) |  |  |
| Yes | 395 (11.7) | 391 (11.9) | 4 (4.1) |  |  | 20 (3.8) | 16 (5.4) | 4 (1.8) |  |  |
| Missing | 0 | 0 | 0 |  |  | 11 (2.1) | 7 (2.3) | 4 (1.8) |  |  |
| **Pleural involvement** |  |  |  | <0.001 | 0.632 |  |  |  | <0.001 | 0.489 |
| No | 2769 (81.8) | 2716 (82.6) | 53 (54.6) |  |  | 358 (68.7) | 232 (77.9) | 126 (56.5) |  |  |
| Yes | 616 (18.2) | 572 (17.4) | 44 (45.4) |  |  | 152 (29.2) | 59 (19.8) | 93 (41.7) |  |  |
| Missing | 0 | 0 | 0 |  |  | 11 (2.1) | 7 (2.3) | 4 (1.8) |  |  |
| **Edge** |  |  |  | <0.001 | 0.407 |  |  |  | 0.886 | 0.044 |
| Smooth | 2254 (66.6) | 2208 (67.2) | 46 (47.4) |  |  | 151 (29.0) | 85 (28.5) | 66 (29.6) |  |  |
| Spiculated | 1131 (33.4) | 1080 (32.8) | 51 (52.6) |  |  | 359 (68.9) | 206 (69.1) | 153 (68.6) |  |  |
| Missing | 0 | 0 | 0 |  |  | 11 (2.1) | 7 (2.3) | 4 (1.8) |  |  |
| **Shape** |  |  |  | 1.000 | 0.009 |  |  |  |  |  |
| Round | 3114 (92.0) | 3025 (92.0) | 89 (91.8) |  |  |  |  |  |  |  |
| Ellipse | 271 (8.0) | 263 (8.0) | 8 (8.2) |  |  |  |  |  |  |  |

**Table S2 Baseline characters of the never-smokers.**

| **Characters** | **Training** set | | | | | **Validation** set | | | | |
| --- | --- | --- | --- | --- | --- | --- | --- | --- | --- | --- |
|  | **Overall** | **Benign** | **Malignant** | ***P*** value | **SMD** | **Overall** | **Benign** | **Malignant** | ***P*** value | **SMD** |
|  | **(N=1780)** | **(N=1728)** | **(N=52)** |  |  | **(N=1282)** | **(N=709)** | **(N=573)** |  |  |
| **Age** (mean±SD) | 57.24±7.80 | 57.20±7.84 | 58.54±6.44 | 0.002 | 0.350 | 51.10±11.17 | 49.85±11.71 | 52.65±10.26 | <0.001 | 0.254 |
| **Sex** |  |  |  | --- | --- |  |  |  | <0.001 | 0.347 |
| Male* | 0 | 0 | 0 |  |  | 750 (58.5) | 443 (62.5) | 307 (53.6) |  |  |
| Female | 1780 (100.0) | 1728 (100.0) | 52 (100.0) |  |  | 300 (23.4) | 180 (25.4) | 120 (20.9) |  |  |
| Missing | 0 | 0 | 0 |  |  | 232 (18.1) | 86 (12.1) | 146 (25.5) |  |  |
| **Education** |  |  |  | 0.987 | 0.023 |  |  |  |  |  |
| Low | 462 (26.0) | 449 (26.0) | 13 (25.0) |  |  |  |  |  |  |  |
| Medium | 1083 (60.8) | 1051 (60.8) | 32 (61.5) |  |  |  |  |  |  |  |
| High | 235 (13.2) | 228 (13.2) | 7 (13.5) |  |  |  |  |  |  |  |
| **Body** mass index (kg/m^2^) |  |  |  | 0.278 | 0.314 |  |  |  |  |  |
| <18·5 | 54 (3.0) | 51 (3.0) | 3 (5.8) |  |  |  |  |  |  |  |
| 18·5-24 | 944 (53.0) | 916 (53.0) | 28 (53.8) |  |  |  |  |  |  |  |
| 24-28 | 641 (36.0) | 621 (35.9) | 20 (38.5) |  |  |  |  |  |  |  |
| ≥28 | 141 (7.9) | 140 (8.1) | 1 (1.9) |  |  |  |  |  |  |  |
| **Frequent** exercise |  |  |  | 0.887 | 0.042 |  |  |  |  |  |
| No | 1299 (73.0) | 1262 (73.0) | 37 (71.2) |  |  |  |  |  |  |  |
| Yes | 481 (27.0) | 466 (27.0) | 15 (28.8) |  |  |  |  |  |  |  |
| **Passive** smoking year |  |  |  | 0.280 | 0.169 |  |  |  |  |  |
| No | 234 (13.1) | 225 (13.0) | 9 (17.3) |  |  |  |  |  |  |  |
| 0-19 | 220 (12.4) | 214 (12.4) | 6 (11.5) |  |  |  |  |  |  |  |
| 20-39 | 1028 (57.8) | 1000 (57.9) | 28 (53.8) |  |  |  |  |  |  |  |
| ≥40 | 298 (16.7) | 289 (16.7) | 9 (17.3) |  |  |  |  |  |  |  |
| **Family** history of lung cancer |  |  |  | 0.340 | 0.159 |  |  |  |  |  |
| No | 642 (36.1) | 627 (36.3) | 15 (28.8) |  |  |  |  |  |  |  |
| Yes | 1138 (63.9) | 1101 (63.7) | 37 (71.2) |  |  |  |  |  |  |  |
| **Chronic respiratory diseases** |  |  |  | 0.280 | 0.169 |  |  |  |  |  |
| No | 95 (5.3) | 90 (5.2) | 5 (9.6) |  |  |  |  |  |  |  |
| Yes | 1685 (94.7) | 1638 (94.8) | 47 (90.4) |  |  |  |  |  |  |  |
| **Emphysema** |  |  |  | 0.717 | 0.079 |  |  |  |  |  |
| No | 1585 (89.0) | 1540 (89.1) | 45 (86.5) |  |  |  |  |  |  |  |
| Yes | 195 (11.0) | 188 (10.9) | 7 (13.5) |  |  |  |  |  |  |  |
| **Maxi**mum diameter(mm) | 9.16±6.50 | 8.95±6.34 | 16.05±7.94 | <0.001 | 0.988 | 11.87±8.76 | 10.92±9.03 | 13.06±8.27 | <0.001 | 0.247 |
| Missing | 0 | 0 | 0 |  |  | 19 (1.5) | 7 (1.0) | 12 (2.1) |  |  |
| **Min**imum diameter(mm) | 7.16±4.84 | 6.99±4.69 | 12.73±6.21 | <0.001 | 1.043 | 9.27±6.90 | 8.58±7.27 | 10.14±6.29 | <0.001 | 0.229 |
| Missing | 0 | 0 | 0 |  |  | 16 (1.2) | 4 (0.6) | 12 (2.1) |  |  |
| **Location** |  |  |  | 0.032 | 0.319 |  |  |  |  |  |
| Not upper lobe | 1093 (61.4) | 1069 (61.9) | 24 (46.2) |  |  |  |  |  |  |  |
| Upper lobe | 687 (38.6) | 659 (38.1) | 28 (53.8) |  |  |  |  |  |  |  |
| **Density** |  |  |  | 0.001 | 0.519 |  |  |  | <0.001 | 0.815 |
| Solid | 1249 (70.2) | 1225 (70.9) | 24 (46.2) |  |  | 579 (45.2) | 428 (60.4) | 151 (26.4) |  |  |
| Part-solid | 293 (16.5) | 277 (16.0) | 16 (30.8) |  |  | 309 (24.1) | 85 (12.0) | 224 (39.1) |  |  |
| Nonsolid | 238 (13.4) | 226 (13.1) | 12 (23.1) |  |  | 351 (27.4) | 175 (24.7) | 176 (30.7) |  |  |
| Missing | 0 | 0 | 0 |  |  | 43 (3.4) | 21 (3.0) | 22 (3.8) |  |  |
| **Calcification** |  |  |  | 0.035 | 0.422 |  |  |  | 0.001 | 0.205 |
| No | 1560 (87.6) | 1509 (87.3) | 51 (98.1) |  |  | 1230 (95.9) | 676 (95.3) | 554 (96.7) |  |  |
| Yes | 220 (12.4) | 219 (12.7) | 1 (1.9) |  |  | 37 (2.9) | 29 (4.1) | 8 (1.4) |  |  |
| Missing | 0 | 0 | 0 |  |  | 15 (1.2) | 4 (0.6) | 11 (1.9) |  |  |
| **Pleural** involvement |  |  |  | <0.001 | 0.551 |  |  |  | <0.001 | 0.439 |
| No | 1475 (82.9) | 1444 (83.6) | 31 (59.6) |  |  | 957 (74.6) | 589 (83.1) | 368 (64.2) |  |  |
| Yes | 305 (17.1) | 284 (16.4) | 21 (40.4) |  |  | 309 (24.1) | 115 (16.2) | 194 (33.9) |  |  |
| Missing | 0 | 0 | 0 |  |  | 16 (1.2) | 5 (0.7) | 11 (1.9) |  |  |
| **Edge** |  |  |  | 0.381 | 0.142 |  |  |  | 0.001 | 0.234 |
| Smooth | 1179 (66.2) | 1148 (66.4) | 31 (59.6) |  |  | 320 (25.0) | 204 (28.8) | 116 (20.2) |  |  |
| Spiculated | 601 (33.8) | 580 (33.6) | 21 (40.4) |  |  | 946 (73.8) | 501 (70.7) | 445 (77.7) |  |  |
| Missing | 0 | 0 | 0 |  |  | 16 (1.2) | 4 (0.6) | 12 (2.1) |  |  |
| **Shape** |  |  |  | 0.250 | 0.248 |  |  |  |  |  |
| Round | 1658 (93.1) | 1607 (93.0) | 51 (98.1) |  |  |  |  |  |  |  |
| Ellipse | 122 (6.9) | 121 (7.0) | 1 (1.9) |  |  |  |  |  |  |  |

*Non-smoking males were defined as low-risk population in the NLCS program.

**Table S3 The comparison between absolute differences between observed and predicted lung cancer risks at the 10^th^, 50^th^, and 90^th^ percentiles for general population in the resampled validation set.**

| **Models** | **10^th^ percentile** | **50^th^ percentile** | **90^th^ percentile** |
| --- | --- | --- | --- |
| Our model | -0.00526 | 0.00399 | -0.01092 |
| McWilliams et al. | -0.00409 | -0.00310 | 0.15135 |
| Tammemagi et al. | 0.26002 | 0.75611 | 0.89074 |
| Gould et al. | -0.00740 | 0.01484 | 0.29873 |
| Zhang et al. | -0.01058 | -0.01766 | 0.08299 |
